# A Multicenter Randomized Trial Assessing ZENFlow Carrier-Free Drug-Coated Balloon for the Treatment of Femoropopliteal Artery Lesions

**DOI:** 10.3389/fcvm.2022.821672

**Published:** 2022-03-15

**Authors:** Leng Ni, Wei Ye, Lan Zhang, Xing Jin, Chang Shu, Jin-song Jiang, Mu Yang, Dan-ming Wu, Ming Li, Guan-feng Yu, Jun Yang, Jian-hua Huang, Xiao-bai Wang, Xiao-qiang Li, Wei-liang Jiang, Zhi-qun Wu, Chang-wei Liu

**Affiliations:** ^1^Department of Vascular Surgery, Peking Union Medical College Hospital, Beijing, China; ^2^Department of Vascular Surgery, Renji Hospital Affiliated to Shanghai Jiaotong University School of Medicine, Shanghai, China; ^3^Department of Vascular Surgery, Shandong Provincial Hospital Affiliated to Shandong First Medical University, Jinan, China; ^4^Vascular Surgery Center, Fuwai Hospital, National Center for Cardiovascular Disease, Beijing, China; ^5^Department of Vascular Surgery, Zhejiang Provincial People's Hospital, Hangzhou, China; ^6^Department of Vascular Surgery, Yantai Yuhuangding Hospital, Yantai, China; ^7^Department of Vascular Surgery, Liaoning Provincial People's Hospital, Shenyang, China; ^8^Department of Vascular Surgery, The First Affiliated Hospital, School of Medicine, Zhejiang University, Hangzhou, China; ^9^Department of Vascular Surgery, The First Affiliated Hospital of Wenzhou Medical University, Wenzhou, China; ^10^Division of Vascular Surgery, Tongji Affiliate Hospital of Tongji Medical College of Huazhong University of Science & Technology, Wuhan, China; ^11^Department of Vascular Surgery, The Xiangya Hospital of Central South University, Shangsha, China; ^12^Interventional Vascular Surgery, The First Affiliated Hospital of Jinan University, Guangzhou, China; ^13^Department of Vascular Surgery, The Second Affiliated Hospital of Soochow University, Suzhou, China; ^14^Department of Vascular Surgery, The Second Affiliated Hospital of Harbin Medical University, Harbin, China; ^15^Interventional Vascular Surgery, Tangdu Hospital, The Second Affiliated Hospital of Air Force Military Medical University, Xian, China

**Keywords:** drug-coated balloon, paclitaxel, percutaneous transluminal angioplasty, restenosis, peripheral artery disease, femoropopliteal artery occlusive disease

## Abstract

**Backgrounds and Objectives:**

Drug-coated balloons (DCBs) have shown promising benefits in improving the outcomes for patients with peripheral artery disease. Several randomized clinical trials have reported that paclitaxel-coated balloon significantly reduce the rates of restenosis and the need for reintervention in comparison with regular balloon angioplasty. Due to the differences in excipients, paclitaxel dose, and coating techniques, variable clinical outcomes have been observed with different DCBs. In this study, we aimed to evaluate the safety and efficacy of a novel ZENFlow carrier-free DCB in the treatment of femoropopliteal artery occlusive disease.

**Methods:**

In this randomized controlled trial conducted at 15 sites, 192 patients with Rutherford class 3–5 were randomly assigned into two groups: drug-coated balloon group and percutaneous transluminal angioplasty group. The primary endpoint was a late lumen loss at 6 months based on blinded angiographic core laboratory evaluations, and the secondary endpoints included primary patency rate, binary restenosis, clinically driven target lesion revascularization, ankle-brachial index, Rutherford class change, and major adverse events.

**Results:**

In this multicenter trial, 93 patients received DCB angioplasty, whereas 99 patients underwent regular balloon angioplasty. The late lumen loss at 6-month follow-up was 0.50 ± 0.82 and 1.69 ± 0.87 mm in the drug-coated balloon and percutaneous transluminal angioplasty groups, respectively (*p* < 0.001). During the 12-month follow-up period, the drug-coated balloon group showed a significantly higher primary patency rate (54 vs. 31.3%, *p* = 0.009) and markedly lower rates of target vessel restenosis (22.1 vs. 64.3%, *p* < 0.001) and clinically driven target lesion revascularization rate (5.4 vs. 19.2%, *p* = 0.006) than the percutaneous transluminal angioplasty group. Compared with the percutaneous transluminal angioplasty group, the drug-coated balloon group had significant improvements in the ankle-brachial index and Rutherford class. The all-cause mortality rate was comparable, and no device-related deaths occurred in either groups.

**Conclusions:**

Balloon angioplasty using a ZENFlow carrier-free drug-coated balloon is a safe and effective treatment method for femoropopliteal artery lesions. This novel drug-coated balloon catheter achieved satisfactory early and 1-year outcomes in this trial.

**Clinical Trial Registration:**

https://clinicaltrials.gov, identifier: NCT03844724.

## Introduction

Superficial femoral and popliteal arteries are the frequently involved arteries in lower extremity ischemia due to atherosclerosis, which is characterized by long and severe calcified lesions with a high incidence of restenosis after percutaneous transluminal angioplasty (PTA) with standard plain balloon catheter. Specifically, in case of flow-limited dissection after PTA, bare metal stent (BMS) implantation can prevent elastic recoil of the injured vessel walls. However, the mid- and long-term outcomes after BMS implantation are not satisfactory because of in-stent restenosis (ISR), stent fractures, and other stent-related complications. According to recent studies, 1-year patency rates after stenting range from 63 to 83% and longer-term patency rates from 60 to 75% (1–5). Thus, investigations on an effective intervention for femoropopliteal artery lesions are ongoing.

Drug-coated balloons (DCBs) have shown benefits in improving long-term patency rate, without the drawbacks of BMS implantations. Most current DCBs are coated with paclitaxel, which can be delivered into the vessel intima during balloon inflation. Based on experimental models, paclitaxel can remain in the vessel wall for up to 180 days and may alleviate intimal hyperplasia after PTA (6). More recently, larger prospective multicenter randomized controlled trials have demonstrated that DCBs have a superior mid-term primary patency rate and are associated with reduced rates of clinically driven target lesion revascularization (CD-TLR) compared with regular PTA (7, 8).

Currently, most DCB catheters using paclitaxel as the drug of choice, but each device differed in terms of the drug dose, selection of the excipient, and overall coating formulation. The ZENFlow paclitaxel-coated balloon (PCB) catheter (Zylox Medical Device Inc., Zhejiang, China) is a carrier-free DCB coated with paclitaxel (3 μg/mm^2^ ± 1 μg/mm^2^) using a unique direct ultrasonic spray coating technique. The paclitaxel coated on the balloon demonstrated a conical microstructure under scanning electron microscopy similar with the original paclitaxel form. According to Chang et al. (9), the conical microstructure can improve the transfer efficacy of paclitaxel into the vessel wall compared with the spherical structure because of the more compressive force onto the arterial wall. In this randomized study, we aimed to evaluate the efficacy and safety of a ZENFlow PCB, a novel carrier-free DCB catheter, in the treatment of femoropopliteal artery occlusive lesions.

## Materials and Methods

### Study Design and Population

In this multicenter, prospective, single-blinded (to patient), randomized (1:1) trial, we compared the early and 1-year outcomes between ZENFlow PCB and an uncoated balloon used in the treatment of femoropopliteal lesions. Randomization was computer generated, and the generated numbers were sealed in envelopes, which were only opened after the evaluation of the target lesions with no flow-limiting dissection based on angiography. The patients and the radiologist/sonographer were blinded to the treatment assignments through the completion of all 12-month follow-up evaluations. Because of the visual difference between the ZENFlow PCB and standard PTA balloon, the treating physicians and catheterization laboratory staff were not blinded to the treatment assignment.

This study was approved by the Peking Union Medical College Hospital Ethics Committee and local Ethics Committees at each trial site. Informed consent was obtained from all patients before study enrollment. The trial was conducted in accordance with the Declaration of Helsinki, Good Clinical Practice guidelines, and provisions for the conduct of clinical trials of medical devices by the China Food and Drug Administration. This trial was registered in the website of ClinicalTrials.gov (Identifier: NCT03844724).

Patients were randomized into two groups: ZENFlow PCB and uncoated regular balloon (PTA) groups. Based on whether patients underwent bail-out stenting or not, subjects in each treatment group were further divided into balloon-only and bail-out stent subgroup. Eligible subjects with 18–85 years of age had severe intermittent claudication or ischemic rest pain or minor tissue loss (Rutherford Clinical Category 3–5); stenosis of 70–99% with lesion lengths ≤30 cm, or a complete occlusion with lengths of ≤10 cm involving the superficial femoral or popliteal arteries (or both). Moreover, patent inflow artery with stenosis ≤30% and at least one run-off infrapopliteal artery were required before enrollment. In patients with occlusive lesions in both limbs, only the lesion on one side leading to more severe symptoms was considered treated. The exclusion criteria were as follows: (1) acute thrombus in the target vessels; (2) severe renal or hepatic dysfunction; (3) known contraindication or allergy to aspirin, clopidogrel, heparin, or paclitaxel; (4) life expectancy <1 year; (5) vessel stenosis or occlusion due to Buerger's disease or autoimmune arteritis; (6) pregnancy; and (7) immunosuppressive agent therapy.

### Procedure and Follow-Up

Dual antiplatelet therapy with aspirin 100 mg/day and clopidogrel 75 mg/day was administered to patients at least 1 week before the procedure, which was continued for at least 3 months after PTA. Unfractionated heparin (80–100 IU/kg, with subsequent boluses adjusted according to the activated clotting time) was administered during the procedure. After successfully crossing the lesion with a guidewire, whether to perform pre-dilation or not was determined by the operator's discretion. When performing pre-dilation, the diameter of standard PTA balloon is usually 1mm less than the reference vessel diameter. The inflation times and pressures were conducted per operator preference. Angiography was performed to re-evaluate the target lesions which received pre-dilation. If no flow-limiting dissections and remaining >30% stenosis in the target lesions were noted, the patients randomly received either ZENFlow PCB catheter or Admiral Xtreme uncoated peripheral balloon catheter (Medtronic, Minneapolis, MN). The balloon catheter size was selected based on the target vessels' reference diameter and target lesions' lengths. Operators were instructed to ensure that the balloon covered the entire pre-dilated segment, to inflate within 3 min of insertion, and to maintain inflation to nominal pressure for at least 1 min. In case of a long lesion requiring multiple DCBs, an overlap of 1 cm was allowed for the adjacent DCBs. Post-dilation or bailout stenting was permitted in either group only for significant (>50%) residual stenosis or total occlusion and flow-limiting dissections. Angiography was performed immediately after the intervention with identical projections (two orthogonal planes for each treated lesion), and the images were compared with the follow-up angiograms.

Clinical follow-up was conducted before discharge from the hospital and at 6 and 12 months after the procedure. Angiography and duplex ultrasound of the target limb were performed at 6 and 12 months, respectively. Rutherford classification and ankle-brachial index (ABI) were evaluated at baseline, before discharge, and at 6 and 12 months. All postoperative clinical events during the 12-month follow-up period were reviewed and evaluated by authorized vascular surgeons, who have pertinent expertise, were not involved in the study, and did not have conflicts of interest, from each medical center.

### Endpoint Definition

Device success was defined as smooth delivering of the balloon catheter to the lesion site, successful balloon inflation and retrieval without rupture during the procedure. Technical success was defined as successful dilation of the target lesions and restoration of blood flow using balloon angioplasty alone, without additional interventions such as stenting or open surgical revascularization.

The primary endpoint was late lumen loss (LLL) at 6 months, which was defined as the difference in minimum lumen diameter of the target lesion between immediately after the intervention and at 6-month follow-up based on angiographic evaluation. Angiographic analyses were performed in an independent core laboratory that was blinded to the treatment assignment; the core laboratory had no information on the treatment arms.

Secondary endpoints included binary restenosis, CD-TLR rate, post-intervention ABI of the target limb and change in Rutherford class at 6 months, primary patency rate and change in Rutherford class at 12 months. Binary restenosis referred to a diameter reduction >50%, which was defined as Doppler ultrasound peak systolic velocity ratio >2.4 at the time of follow-up. CD-TLR was defined as any reintervention of the target lesions due to clinically indicated reoccurrence of target limb ischemic symptoms, as adjudicated by the clinical-events committee, and a target lesion diameter stenosis of ≥70% detected by angiography or duplex ultrasound. The primary patency was defined as the absence of binary restenosis and freedom from CD-TLR. Major adverse events (MAEs) were defined as all-cause death, major amputation of the target limb, or target vessel revascularization (TVR) during the 12 months' follow-up.

### Statistical Analysis

To provide the study with a 90% power to detect LLL differences at 6 months (with an expected mean LLL of 0.46 ± 1.13 mm in the ZENFlow PCB group and 1.09 ± 1.07 mm in the PTA group) at a two-sided α = 0.05, the calculated sample size was 64 patients per group. All analyses were based on the intention-to-treat principle. Continuous variables were described as mean ± standard deviation and dichotomous and categorical variables as numbers and proportions. For treatment group comparisons, *t*-test or Wilcoxon rank-sum test was used to process continuous data and Pearson χ^2^ test or Fisher exact test was used to analyze categorical data, as appropriate. A multivariable linear regression analysis to identify independent predictors of primary endpoints was performed. Moreover, the Kaplan-Meier method was employed to evaluate time-to-event data for freedom from CD-TLR during the 12-month follow-up period. Difference in the survival curves between groups was assessed using the log-rank test. All statistical analyses were performed with a two-sided significance level of 0.05. Statistical analysis was performed using SAS (SAS Institute, Cary, NC) version 9.1 or higher.

## Results

### Baseline Patient and Lesion Characteristics

**Figure 1:**
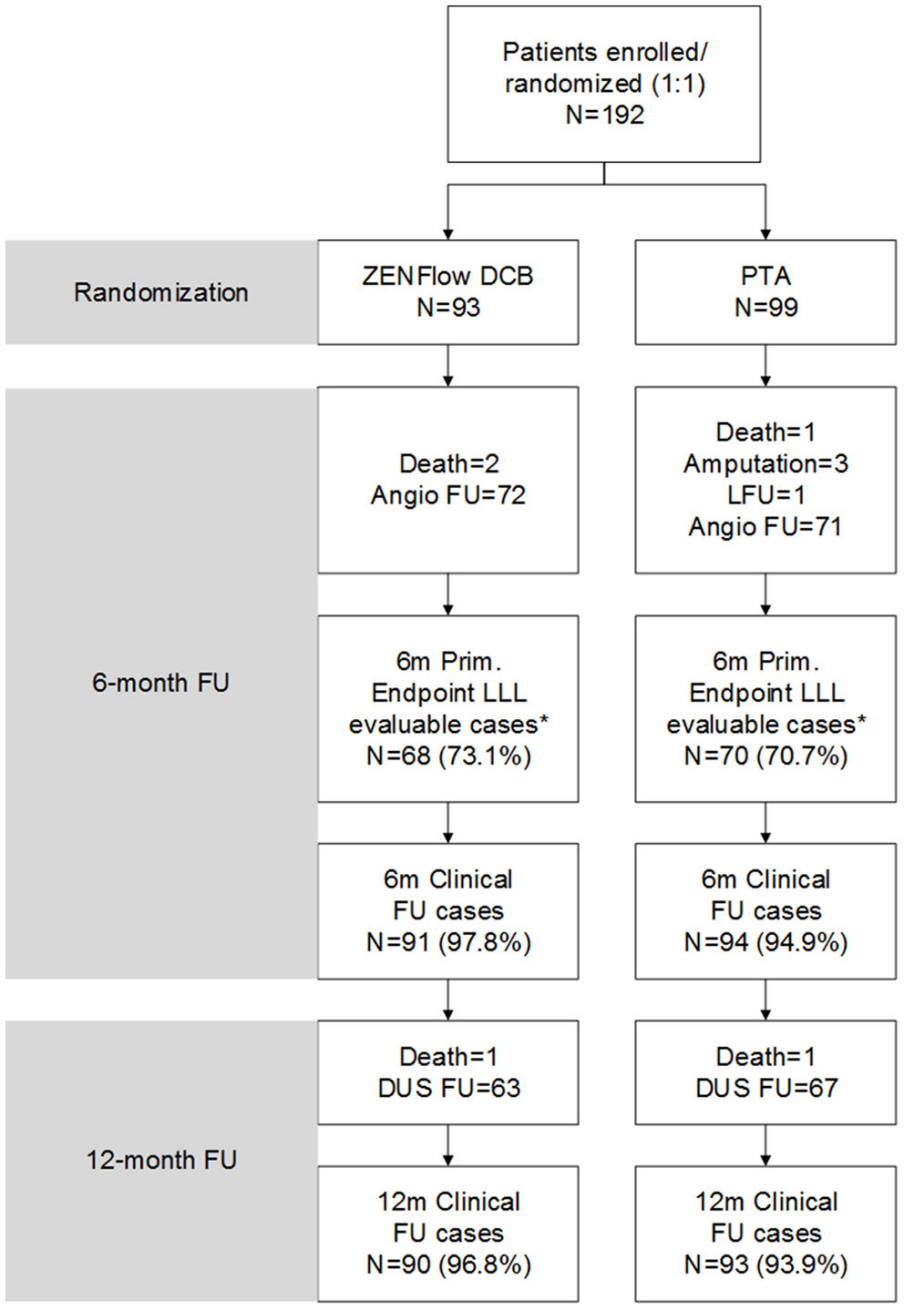
Flow diagram of patients. *Four patients in the DCB group and one patient in the PTA group underwent angiography at 6 months but did not match the imaging quality for LLL evaluation. DCB, drug-coated balloon; DUS, duplex ultrasound; FU, follow-up; LFU, loss to follow-up; LLL, late lumen loss; PTA, percutaneous transluminal angioplasty.

In this multicenter trial, a total of 192 patients (192 lesions) were recruited and randomized; 93 received ZENFlow PCB and 99 received uncoated Admiral Xtreme balloon (Figure 1). No significant difference in patient demographics, comorbidities, baseline ABI, and Rutherford class was found between the DCB and PTA groups (Table 1). Most of the target lesions involved the superficial femoral artery and were de novo lesions in both groups. The DCB group had longer target lesions than the PTA group (70.3 ± 63.3 mm vs. 54.6 ± 54.1 mm), although the difference was not significant (*p* = 0.07). The proportion of total occlusive lesions was significantly lower in the DCB group than in the PTA group (29 vs. 44.4%, *p* = 0.04).

**Table 1:** Patient demographics and lesion characteristics.

	**DCB group**	**PTA group**	***P-*value**
	**(*n* = 93)**	**(*n* = 99)**	
Age, years	68.8 ± 8.3	68.1 ± 10.5	0.61
Male	67 (72.0)	71 (71.7)	0.96
Diabetes	46 (49.5)	46 (46.5)	0.57
Hypertension	63 (67.7)	68 (68.7)	0.48
Hyperlipidemia	26 (28.0)	22 (22.2)	0.52
Current smoker	47 (50.5)	48 (48.5)	0.78
Ankle-brachial index, baseline	0.57 ± 0.28	0.59 ± 0.34	0.74
Baseline Rutherford category			0.87
3	35 (67.7)	37 (67.7)	
4	17 (18.3)	22 (22.2)	
5	13 (14.0)	10 (10.1)	
Target lesion location			0.26
SFA	81 (87.1)	77 (77.8)	
Popliteal artery	9 (9.7)	16 (16.2)	
SFA + popliteal artery	3 (3.2)	6 (6.1)	
Target lesion type			0.55
De novo	80 (86.0)	88 (88.9)	
In-stent restenosis	13 (14.0)	11 (11.1)	
Lesion length, mm	70.3 ± 63.3	54.6 ± 54.1	0.07
Total occlusions, *n* (%)	27 (29.0)	44 (44.4)	0.04
Severe calcification, *n* (%)	46.2 (43)	40.4 (40)	0.47

*Values are mean ± SD or n (%). DCB, drug-coated balloon; PTA, percutaneous transluminal artery; SFA, superficial femoral artery*.

### Procedure Information

Details of the procedures and perioperative outcomes in both groups are described in Table 2. Pre-dilations were performed in the majority of patients in both groups. The number of balloons per case was greater in the DCB than in the PTA group because DCB could only be used once, whereas uncoated balloons could be repeatedly inflated in cases with long lesions beyond the length of a single balloon. Although the PTA group presented a non-significant increase in the degree of in-segment diameter stenosis compared with the DCB group (86.3 ± 15.5% vs. 81.8 ± 16.7%, *p* = 0.052), the degree of post-dilation residual stenosis of the target lesion was comparable between the groups (11.6 ± 12.1% vs. 12.4 ± 13.2%, *p* = 0.63). Bailout stenting occurred more frequently in the PTA group than in the DCB group (17.2 vs. 6.5%, *p* = 0.02). Technical success was significantly higher in the DCB group than in the PTA group (95.7 vs. 83.8%, *p* = 0.007). One patient from the DCB group was diagnosed with myocardial ischemia and severe hypotension, which were identified as serious adverse events, within 30 days postoperatively.

**Table 2:** Procedure information.

	**DCB group**	**PTA group**	***P-*value**
	**(*n* = 93)**	**(*n* = 99)**	
Pre-dilation	91 (97.9)	98 (99.0)	0.61
Study balloon deployment			
Number of balloons	1.3 ± 0.6	1.1 ± 0.3	0.0003
Inflation duration (sec)	151.8 ± 50.1	143.5 ± 48.6	0.25
MLD pre-procedure (mm)^*^	0.9 ± 0.9	0.7 ± 0.8	0.06
Reference vessel diameter (mm)^*^	4.9 ± 0.9	4.8 ± 0.9	0.51
In-segment diameter stenosis, %	81.8 ± 16.7	86.3 ± 15.5	0.052
Post-PTA target lesion minimal diameter, mm^*^	4.3 ± 1.1	4.2 ± 1.0	0.63
Post-PTA reference vessel diameter, mm^*^	4.8 ± 0.9	4.8 ± 0.9	0.98
Residual stenosis, %	11.6 ± 12.1	12.4 ± 13.2	0.63
Bailout stenting, *n* (%)	6 (6.5)	17 (17.2)	0.02
Procedural outcome			
Device success	93 (100)	99 (100)	NA
Technical success	89 (95.7)	83 (83.8)	0.007
SAE through discharge	1 (1.1)	0 (0)	0.48

* *Quantitative vascular angiography measurements. DCB, drug-coated balloon; PTA, percutaneous transluminal artery; MLD, minimal luminal diameter; SAE, serious adverse events*.

### Treatment Outcomes

**Figure 2:**
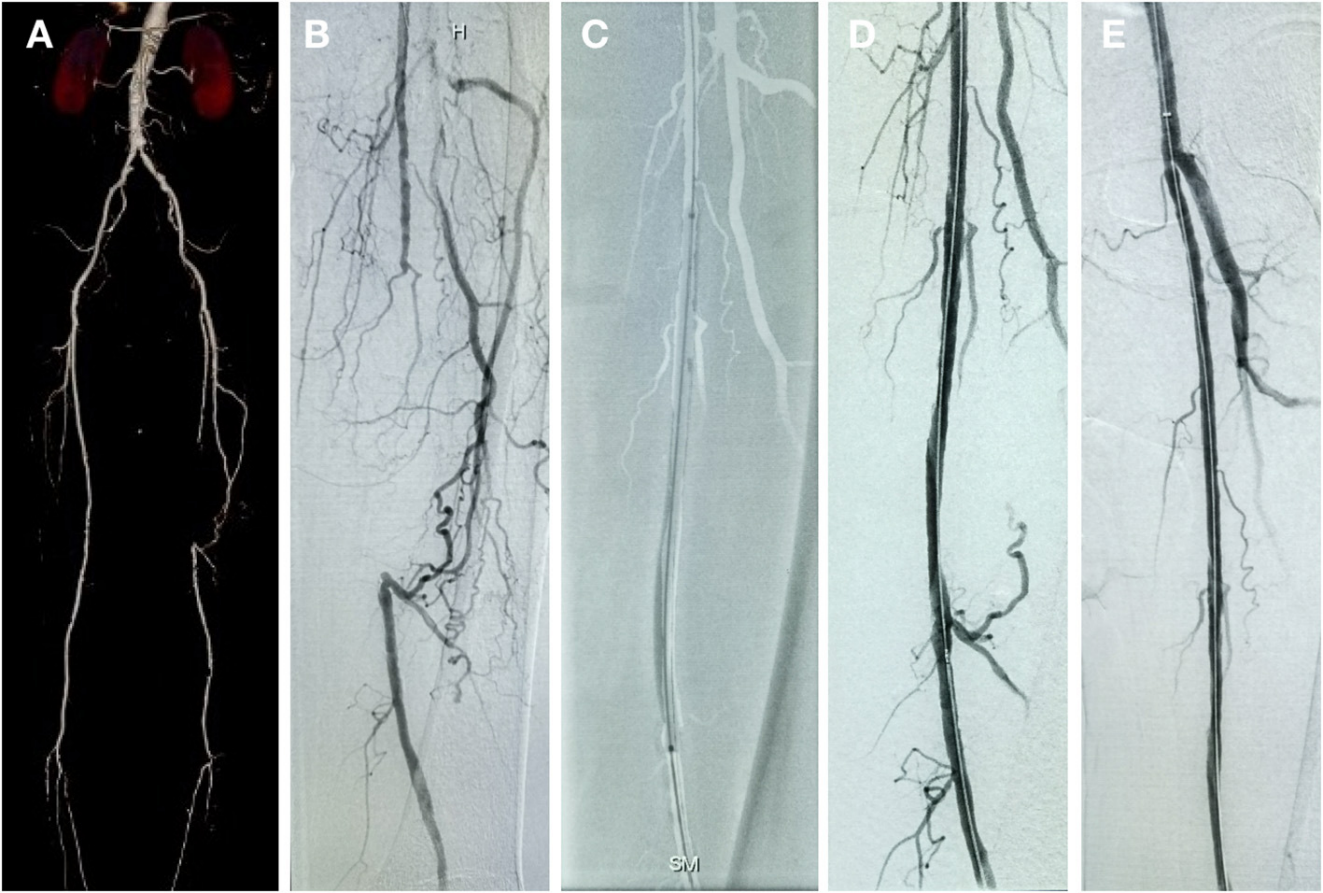
Patient with chronic total occlusive lesion in the left SFA treated with DCB and follow-up result. **(A)** Preprocedure computed tomography angiography (CTA). **(B)** Initial angiogram during procedure. **(C)** Inflated balloon. **(D)** Postprocedure angiogram. **(E)** 6-month follow-up angiogram showing no restenosis of the target lesion.

**Figure 3:**
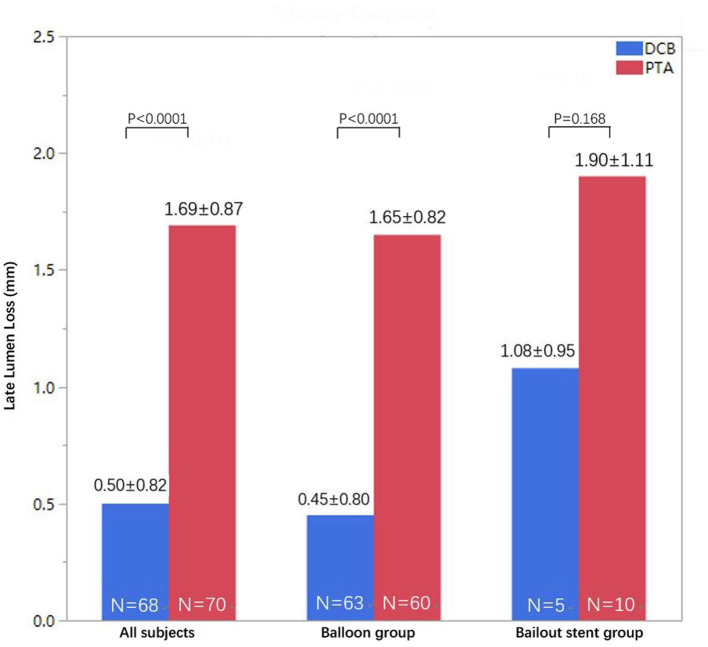
Distribution of LLL at 6 months in the DCB and PTA groups in the intention-to-treat population (all subjects in pooled strata) and separately for each stratum (intended balloon or bailout stent groups). DCB, drug-coated balloon; LLL, late lumen loss; PTA, percutaneous transluminal angioplasty.

**Figure 4:**
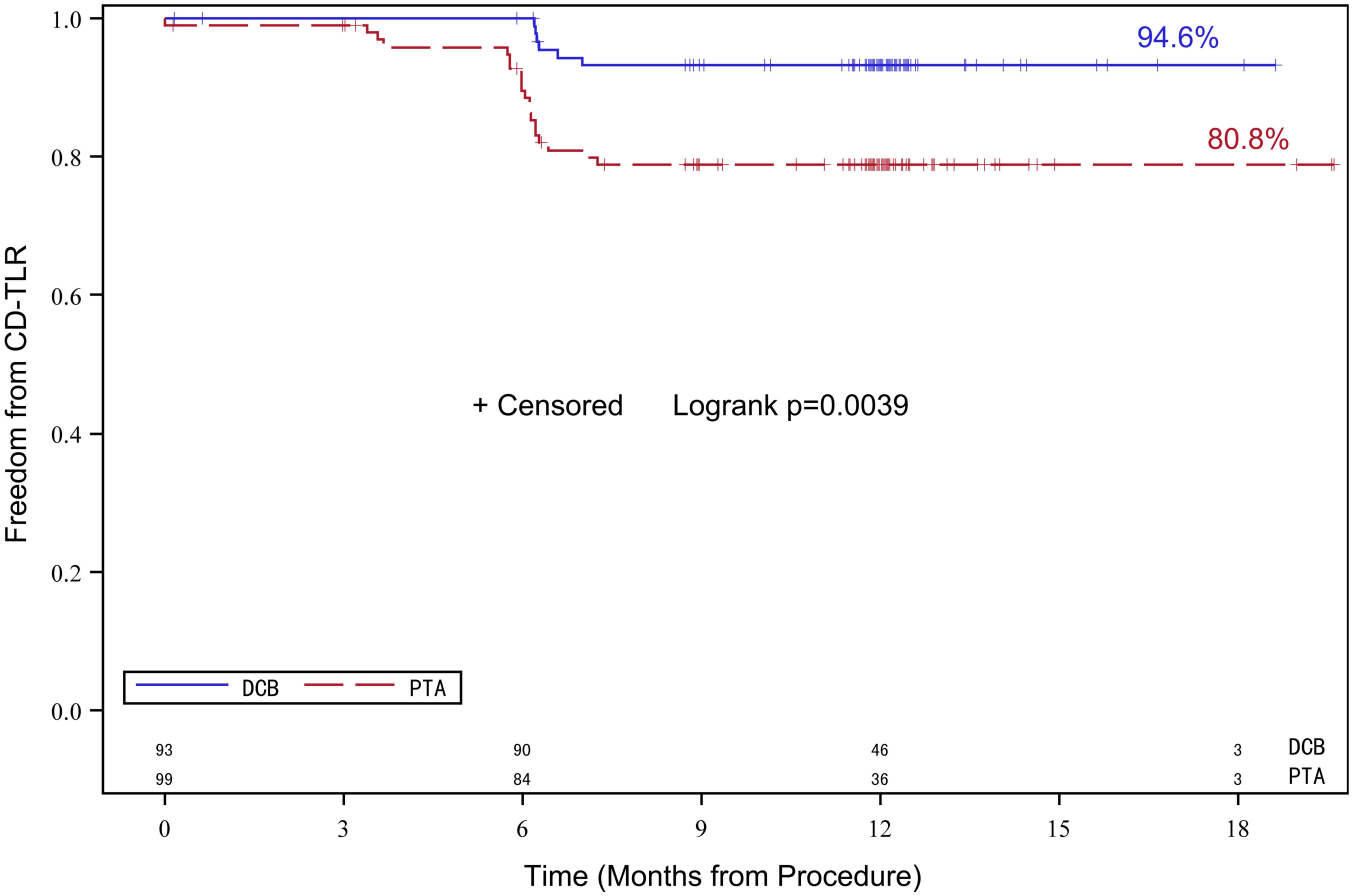
Kaplan-Meier survival analysis of freedom from CD-TLR. Data of up to 180 days post-treatment are shown. CD-TLR, clinically driven target lesion revascularization.

The number of patients who withdrew consent or were not available for angiography at 6 months was comparable in both treatment groups. Moreover, 72 patients in the DCB group and 71 patients in the PTA group underwent angiographic evaluation at 6-month follow-up (Figure 2). Duplex ultrasound follow-up results were obtained in 63 and 67 patients in the DCB group and PTA group at 12 months, respectively. The LLL at 6 months was significantly lower in the DCB group than in the PTA group (0.50 ± 0.82 mm vs. 1.69 ± 0.87 mm, *p* < 0.0001). The LLL in the balloon-only stratum was also significantly lower in the DCB group than in the PTA group (0.45 ± 0.80 mm vs. 1.65 ± 0.82 mm, *p* < 0.0001). No significant difference was observed in the LLL in the bail-out stenting stratum (DCB group at 1.08 ± 0.95 mm vs. PTA group at 1.90 ± 1.11 mm, *p* = 0.168) (Figure 3). Subgroup analyses on age (<75 years vs. ≥75 years), diabetes vs. non-diabetes, de novo vs. restenotic lesions, and relatively short (<10 cm) vs. long stenotic lesions (≥10 cm) were performed, and the results confirmed the significant superiority of DCB (Supplementary Figure 1). Considering the unbalanced distribution of the lesion length, proportion of the total occlusive lesion, and bail-out stenting rate in the two groups, a multivariable linear regression analysis was performed to identify independent predictors of primary endpoints. After adjustment for other potential confounders, only DCB use was associated with significant decrease in LLL (Supplementary Table 1). Furthermore, the DCB group demonstrated better outcomes in terms of primary patency rate at 12 months (54 vs. 31.3%, *p* = 0.009) and binary restenosis rate at 6 months (22.1 vs. 64.3%, *p* < 0.001) than the PTA group. With regard to the total occlusive lesions, we also found the DCB group had higher 12-month patency rate than the PTA group (60 vs. 31%, *p* = 0.064); due to the limited number of patients received follow-up evaluation, no significant difference was identified. Freedom from CD-TLR by Kaplan-Meier estimate was significantly higher in the DCB group than in the PTA group (94.6 vs. 80.8%; log-rank *p* = 0.004) at 6 months (Figure 4). The mean ABI of the target limb at 6 months after the procedure was distinctly higher in the DCB group than in the PTA group (0.89 ± 0.27 vs. 0.78 ± 0.28, *p* = 0.02); both groups had similar baseline ABI. Additionally, the distribution of patients with respect to Rutherford class improvement was significantly different between the DCB and PTA groups at 6 months (*p* = 0.04); however, no significant difference at 12 months was found (*p* = 0.9) (Table 3).

**Table 3:** Treatment outcomes.

	**DCB group**	**PTA group**	***P*-value**
	**(*n* = 93)**	**(*n* = 99)**	
LLL at 6 months (mm)	0.50 ± 0.82 (68)	1.69 ± 0.87 (70)	<0.0001
Primary patency rate at 12 months, % (m/n)	54.0 (34/63)	31.3 (21/67)	0.009
Binary restenosis rate at 6 months, % (m/n)	22.1 (15/68)	64.3 (45/70)	0.0007
CD-TLR at 6 months, % (m/n)	5.4 (5/93)	19.2 (19/99)	0.0062
Post-PTA ABI at 6 months	0.89 ± 0.27 (72)	0.78 ± 0.28 (71)	0.02
Improvement of ABI from baseline	0.29 ± 0.30	0.12 ± 0.39	0.01
Improvement of Rutherford category from baseline at 6 months	*N* = 72	*N* = 71	0.04
<0	63 (87.5)	55 (77.5)	
=0	9 (12.5)	10 (14.1)	
>0	0 (0)	6 (8.5)	
Improvement of Rutherford category from baseline at 12 months	*N* = 64	*N* = 63	0.90
<0	53 (82.8)	52 (82.5)	
=0	8 (12.5)	7 (11.1)	
>0	3 (4.7)	4 (6.4)	
MAE at 12 months	11 (11.8)	25 (25.3)	0.03
All-cause mortality at 12 months	3 (3.2)	2 (2.0)	0.67
Device- and procedure-related death	0 (0)	0 (0)	NA
TVR at 12 months	8 (8.6)	23 (23.2)	0.006
Target limb major amputation at 12 months	0 (0)	1 (1.0)	0.52

*Values are mean ± SD or n (%). ABI, ankle-brachial index; CD-TLR, clinically driven target lesion revascularization; DCB, drug-coated balloon; LLL, late lumen loss; m, numbers in category; MAE, major adverse events; n, number of available values; PTA, percutaneous transluminal angioplasty; TVR, target vessel revascularization*.

MAEs were reported in 18.8% (36/192) of all patients. The DCB group had a significantly lower MAE rate than the PTA group (11.8 vs. 25.3%, *p* = 0.03). The all-cause mortality rate was 2.6% (5/192 patients). No device- or procedure-related deaths were observed. No significant difference in the mortality rates was noted between the groups (3.2 vs. 2.0%, *p* = 0.67). The DCB group displayed substantial advantages in the TVR rate compared with the PTA group (8.6 vs. 23.2%, *p* = 0.006). One patient from the PTA group had major target limb amputation due to deterioration of limb ischemia.

## Discussion

This randomized study investigated the efficacy and safety of ZENFlow carrier-free PCB catheters in the treatment of atherosclerotic femoropopliteal lesions. The results demonstrated the superiority of the ZENFlow PCB in early and 1-year outcomes over the standard PTA, with a 70% reduction in 6-month LLL for the ZENFlow PCB group (0.50 mm) when compared with the uncoated balloon control group (1.69 mm).

Superficial femoral and popliteal arteries are the commonly involved blood vessels in patients with peripheral artery disease. Over the past few decades, the standard PTA with or without stent implantation is the first-line treatment option. However, ISR following stenting occurs and thus has become the primary issue, resulting in failure of the interventions. The ISR rate after stenting in the femoropopliteal artery was reported as 30–40% within 2–3 years after implantations (10–12). Recently, DCB has been an attractive alternative because it offers the promise of improved patency compared with standard PTA and avoids ISR occurrence after treatment with stents. Well-conducted randomized controlled trials have provided solid evidence of the superiority of DCBs over PTA with uncoated balloons. Existing data from these trials showed significant reductions in LLL, higher primary patency rate, and lower CD-TLR rates during the follow-up period with DCB than with PTA (13–15).

Although most of the marketed devices thus far have used paclitaxel, all use different drug carriers, each of which can vary widely in its transfer efficiency. The ideal DCB should rapidly and efficiently deliver a therapeutic dose to the vessel wall with the lowest possible drug load on the balloon to minimize the downstream drug effect. The most unique characteristic of the ZENFlow PCB catheter used in this study is its novel carrier-free coating technology. The ultrasonic spray coating technique ensures a uniform and stable conical-shape paclitaxel loading, which in turn facilitates efficient and rapid drug delivery into the vessel wall after balloon inflation. In addition, sparing carrier-coating can help avoid possible interference and systemic side-effects derived from the excipients.

To date, this is the first report on the treatment of femoropopliteal artery atherosclerotic lesions using carrier-free DCB catheter in Chinese population. Notably, due to the different enrollment criteria, baseline characteristics of patients and target lesions, procedure protocols, primary endpoint and evaluation methods, clinical outcomes may differ across studies. The LLL, the primary endpoint of this study, was significantly decreased in the DCB group compared with the PTA group, which is consistent with the findings of other trials that have also used LLL as the primary endpoint (7, 15, 16). A *post-hoc* subgroup analysis demonstrated favorable outcomes of DCB across various clinical and anatomic subgroups, including patients with diabetes and longer occlusive lesions. This finding was confirmed by the significant improvement in secondary endpoints, including binary restenosis, primary patency rate, CD-TLR, and ABI and Rutherford class at 6 and 12 months, in the DCB group compared with the PTA group. The rate of freedom from CD-TLR at 6 months was 94.6% for the DCB group and 80.8% for the PTA group. The primary patency rate at 12 months was higher in the DCB group than in the PTA group (54 vs. 31.3%). However, the primary patency rates in the two treatment groups in this trial were relatively lower than those in other randomized trials (14, 17). This discrepancy is mostly attributable to the relatively low follow-up rate in our study. Some of the asymptomatic patients whose target lesions might be patent were reluctant to receive duplex ultrasound follow-up at 12 months. Therefore, these patients may have reduced the overall total primary patency rate. The results of this study are in good agreement with those of the recently published AcoArt I Trial (15), which had a similar study design. In both studies, the primary endpoint, i.e., LLL after 6 months, was significantly lower in patients treated with a paclitaxel-coated balloon; however, the mean LLL in the DCB group in our study was higher than that in the AcoArt I trial (0.50 ± 0.82 mm vs. 0.05 ± 0.73 mm). Moreover, bailout stentings were performed more frequently in the PTA group than in the DCB group, which could be explained by the higher number of total occlusive lesions, resulting in more treatment failures, in the PTA group. In addition, ZENFlow PCB was associated with a low MALE rate. No target vessel thrombosis, which is a typical concern in local vascular delivery, was observed in the DCB group.

In a previous meta-analysis, Katsanos et al. demonstrated a significantly increased risk of death with the use of paclitaxel-coated balloons and stents in the femoropopliteal arteries (18). The 3-year results of the recently published the IN.PACT trial also showed that the all-cause mortality rate was significantly higher in the DCB group than in the PTA group (8). Thus, the most common concern about paclitaxel-coated DCB is long-term mortality. In our study, no serious adverse events associated with the paclitaxel-coated balloon were observed. However, due to limited follow-up duration, the paclitaxel- associated mortality could not be fully determined in current study. Long-term follow-up results are expected for further investigation on the mortality in the patients with paclitaxel-coated DCB treatment.

### Study Limitations

This study has several limitations. First, the number of patients who agreed to undergo angiography at 6 months and duplex ultrasound at 12 months was less than optimal, which possibly affected the comparisons of primary endpoint LLL and long-term follow-up outcomes, especially the lower limb functional outcomes such as walking distance treadmill assessment. Second, the higher prevalence of longer lesion length in the DCB group and total occlusive lesions in the PTA group may have influenced the study results. For example, bailout stenting for treatment of flow-limiting dissection was more frequent for the control than the DCB group. Moreover, owing to the differences in the appearance of balloon coatings, blinding of the investigators was not completely feasible during the study. The physicians responsible for the clinical follow-up of the patients were also not blinded to the treatment received, which might have influenced the clinical decision-making. Finally, the relatively short follow-up duration is a further limitation of this study. A longer-term follow-up is needed to further confirm the durability of the DCB benefits.

## Conclusions

In conclusion, in this prospective, multicenter, randomized trial, the novel ZENFlow PCB was superior to standard PTA and had a favorable safety profile in patients with symptomatic femoropopliteal artery disease.

## Data Availability Statement

The raw data supporting the conclusions of this article will be made available by the authors, without undue reservation.

## Ethics Statement

The studies involving human participants were reviewed and approved by Peking Union Medical College Hospital Ethics Committee and Local Ethics Committees at each trial site. The patients/participants provided their written informed consent to participate in this study.

## Author Contributions

C-wL and CS: conception and design and overall responsibility. LN, C-wL, CS, WY, and LZ: analysis and interpretation. WY, LZ, XJ, CS, J-sJ, MY, D-mW, ML, G-fY, JY, J-hH, X-bW, X-qL, W-lJ, and Z-qW: data collection. LN and WY: writing the article and statistical analysis. LN, LZ, XJ, CS, J-sJ, MY, D-mW, ML, G-fY, JY, J-hH, X-bW, X-qL, W-lJ, Z-qW, and C-wL: critical revision of the article. All authors read and approved the final version of the manuscript.

## Conflict of Interest

The authors declare that the research was conducted in the absence of any commercial or financial relationships that could be construed as a potential conflict of interest.

## Publisher's Note

All claims expressed in this article are solely those of the authors and do not necessarily represent those of their affiliated organizations, or those of the publisher, the editors and the reviewers. Any product that may be evaluated in this article, or claim that may be made by its manufacturer, is not guaranteed or endorsed by the publisher.

## Supplementary Material

Supplementary Figure 1 Subgroup analyses for LLL at 6 months. DCB, drug-coated balloon; PTA, percutaneous transluminal angioplasty.

## Abbreviations

DCB: drug-coated balloon
PCB: paclitaxel-coated balloon
PTA: percutaneous transluminal angioplasty
ISR: in-stent restenosis
LLL: late lumen loss
ABI: ankle-brachial index
CD-TLR: clinically driven target lesion revascularization
TVR: target vessel revascularization
MAE: major adverse event(s).
