# Supplementary figures and images for: A Multicenter Randomized Trial Assessing ZENFlow Carrier-Free Drug-Coated Balloon for the Treatment of Femoropopliteal Artery Lesions
Source: Front Cardiovasc Med. 2022 Mar 15;9:821672. doi: 10.3389/fcvm.2022.821672 (PMC8982076; doi:10.3389/fcvm.2022.821672)

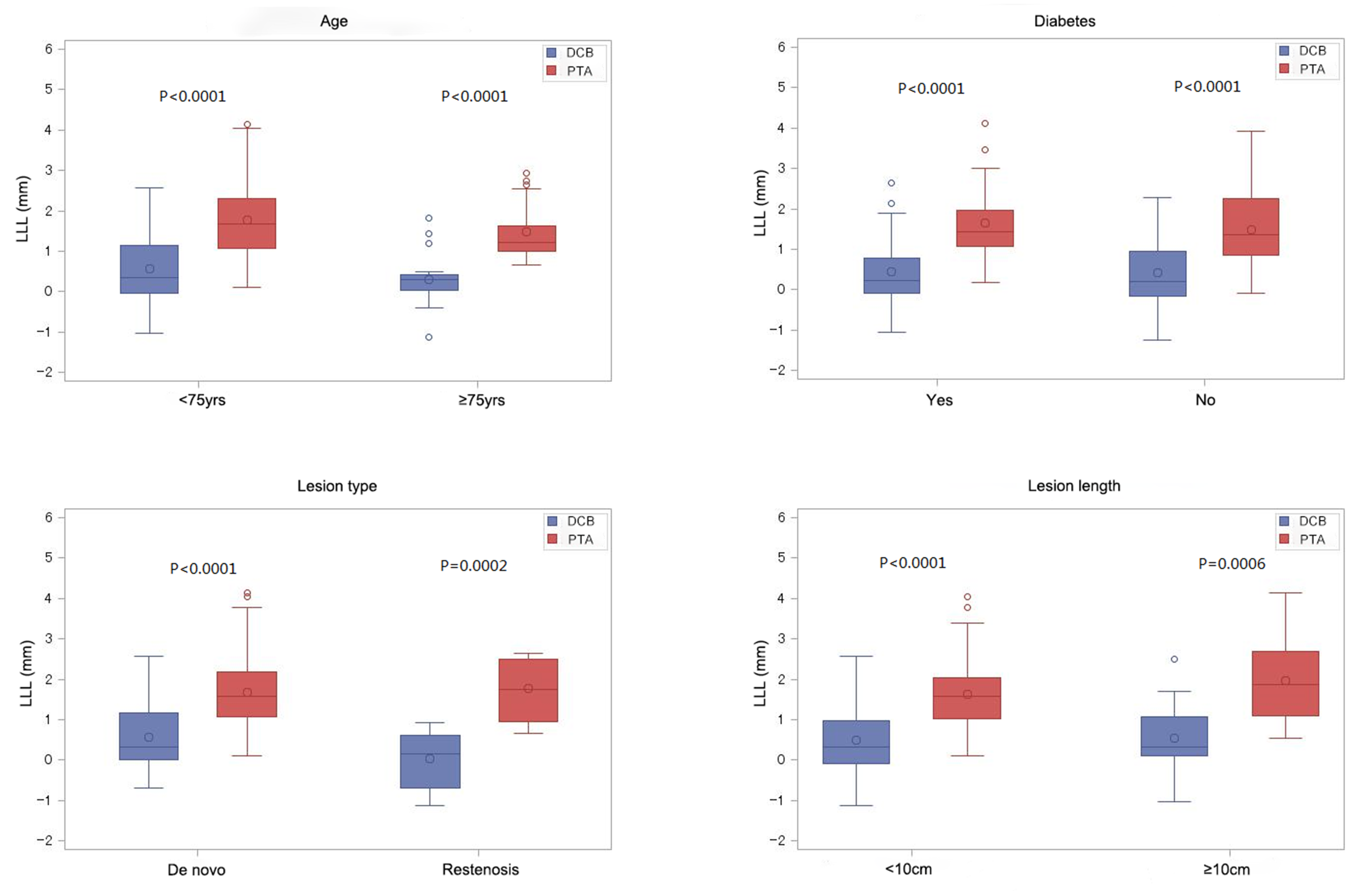

Supplement: Supplementary Figure 1 — Subgroup analyses for LLL at 6 months. DCB, drug-coated balloon; PTA, percutaneous transluminal angioplasty. [file Image_1.TIF]
