# Supplementary material for: A Multicenter Randomized Trial Assessing ZENFlow Carrier-Free Drug-Coated Balloon for the Treatment of Femoropopliteal Artery Lesions
Source: Front Cardiovasc Med. 2022 Mar 15;9:821672. doi: 10.3389/fcvm.2022.821672 (PMC8982076; doi:10.3389/fcvm.2022.821672)
Supplement: Supplementary file 2 [file Table_1.DOCX]

**Supplementary Table 1. Multiple linear regression analysis for LLL**

| **Variable** | **Parameter Estimate** | **Standard**  **Error** | **t Value** | **P Value** |
| --- | --- | --- | --- | --- |
| DCB use | -1.17955 | 0.15741 | -7.49 | <.0001 |
| Lesion length | 0.00281 | 0.00163 | 1.73 | 0.0872 |
| Bailout-stent | 0.42181 | 0.27115 | 1.56 | 0.1228 |
| Total occlusion | 0.35113 | 0.18311 | 1.92 | 0.0578 |

DCB, drug-coated balloon
